# Application of M13 phage display for identifying immunogenic proteins from tick (*Ixodes scapularis*) saliva

**DOI:** 10.1186/s12896-015-0167-3

**Published:** 2015-05-30

**Authors:** Martin Becker, André Felsberger, André Frenzel, Wendy M. C. Shattuck, Megan Dyer, Jonas Kügler, Jonas Zantow, Thomas N. Mather, Michael Hust

**Affiliations:** Institut für Biochemie, Biotechnologie und Bioinformatik, Technische Universität Braunschweig, Spielmannstr.7, 38106 Braunschweig, Germany; University of Rhode Island, URI Center for Vector-Borne Disease, 231 Woodward Hall, 9 East Alumni Avenue, Suite 7, 02881 Kingston, RI USA; Present Address: Max-Planck-Institute for Immunobiology and Epigenetics, Stuebeweg 51, 79108 Freiburg, Germany; Present Address: YUMAB GmbH, Rebenring 33, 38106 Braunschweig, Germany

## Abstract

**Background:**

Ticks act as vectors for a large number of different pathogens, perhaps most notably *Borrelia burgdorferi,* the causative agent of Lyme disease. The most prominent tick vector in the United States is the blacklegged tick, *Ixodes scapularis*. Tick bites are of special public health concern since there are no vaccines available against most tick-transmitted pathogens. Based on the observation that certain non-natural host animals such as guinea pigs or humans can develop adaptive immune responses to tick bites, anti-tick vaccination is a potential approach to tackle health risks associated with tick bites.

**Results:**

The aim of this study was to use an oligopeptide phage display strategy to identify immunogenic salivary gland proteins from *I. scapularis* that are recognized by human immune sera. Oligopeptide libraries were generated from salivary gland mRNA of 18 h fed nymphal *I. scapularis*. Eight immunogenic oligopeptides were selected using human immune sera. Three selected immunogenic oligopeptides were cloned and produced as recombinant proteins. The immunogenic character of an identified metalloprotease (MP1) was validated with human sera. This enzyme has been described previously and was hypothesized as immunogenic which was confirmed in this study. Interestingly, it also has close homologs in other *Ixodes* species.

**Conclusion:**

An immunogenic protein of *I. scapularis* was identified by oligopeptide phage display. MP1 is a potential candidate for vaccine development.

## Background

Pathogen transmission by ticks is a worldwide problem that causes significant health risks and economical losses [1]. The deer tick, *Ixodes scapularis,* which is prevalent in the eastern and upper mid-western United States, functions as a vector for a number of pathogens including the Lyme disease spirochete, *Borrelia burgdorferi,* [2] that the U.S. Centers for Disease Control estimates currently causes 300,000 new infections each year solely in the United States [3, 4]. To date, preventing tick-transmitted diseases relies mostly on the application of acaricides and protecting against tick bites [5]. Currently, no vaccines are available for most tick-transmitted pathogens, emphasizing the health risks posed by tick-bites. Pathogen transmission is substantially aided by bioactive components of tick saliva providing anti-inflammatory or anti-haemostatic effects amongst other activities [6–8]. Several studies have demonstrated the importance of specific tick salivary proteins in pathogen transmission [2, 9]; pathogen transmission from tick vectors is significantly more efficient compared to pathogen transmission through a needle and syringe, underlining the importance of tick saliva in this process [10].

It is noteworthy that the transcriptome of tick salivary glands is highly dynamic and changes rapidly during feeding [11]. Even more interesting is the observation that early salivary proteins, those expressed within the first 24 hours of feeding, appear to be sufficient to induce an immune response in certain hosts [12] while during this early phase of blood feeding, few if any pathogens are transferred from tick to host [2].

Certain host animals can acquire immunity to tick bites after repeated infestations [13–15]. Acquired tick resistance (ATR) and the tick protective response can manifest itself in many different ways. Among the valid definitions listed in the literature are decreased numbers of ticks successfully engorging on a host, premature tick detachment, decreased oviposition or even death of the tick as well as host hypersensitivity reactions [15, 16]. A study focusing on basophils and mast cells as well as different Ig receptors on their surface showed that these cell types as well as immunoglobulins of the IgG and the IgE class are essential for acquired tick immunity [17]. Furthermore, acquired tick immunity also has been correlated with impaired pathogen transmission [18, 19], inspiring the concept of anti-tick vaccination. Such a strategy could potentially alleviate human health risks by preventing infection with a number of pathogens through a single, vector-targeted approach to vaccination. Several studies have begun assessing this idea, using serum antibodies from tick sensitized animals as probes for identifying immunogenic proteins in tick saliva, followed by testing the vaccination potential of the identified antigens in immunization studies [20, 21]. However, to our knowledge there is no study identifying tick salivary antigens as potential vaccine candidates that are recognized specifically by humans.

Phage display is a robust technique suited for high-throughput screening of specific and high-affinity interactions between biomolecules, especially proteins and peptides such as antibodies and/or antigens [22–24]. Phage particles can directly be engineered to display foreign proteins on their surface while carrying the genetic information inside their capsid, thus providing an intrinsic connection of genotype and phenotype [25, 26]. Phage display libraries are readily generated from genomic DNA of prokaryotes and cDNA of eukaryotes. A major burden during library construction is the high rate of DNA fragments cloned out of frame with the phage coat protein to be fused to for display [27]. This pitfall is significantly relieved by the pHORF/Hyperphage system, which includes an ORF enrichment step, improving the efficiency of library packaging and subsequent screening [28–31]. An overview about M13 phage display derived technologies for selecting immunogenic proteins/biomarkers is given elsewhere [32].

In this study a phage display antigen library composed of cDNA derived from 18 h fed nymphal *I. scapularis* salivary gland mRNA was generated. The goal was to screen this library against IgG antibodies from human donors that self-identified as having strong reactions to tick bites, including redness and itching, pre-mature tick detachment or even death of the tick. Proteins identified in this screen are proposed to be useful in at least two ways. First, these proteins might serve as future vaccine candidates to immunize against tick bites and in this way to prevent tick transmitted diseases. Second, the presence of antibodies against these identified proteins may be useful as biomarkers of tick exposure or recent tick bites.

## Results

### Construction of an antigen library from salivary glands of *I. scapularis* nymphs

**Fig 1:**
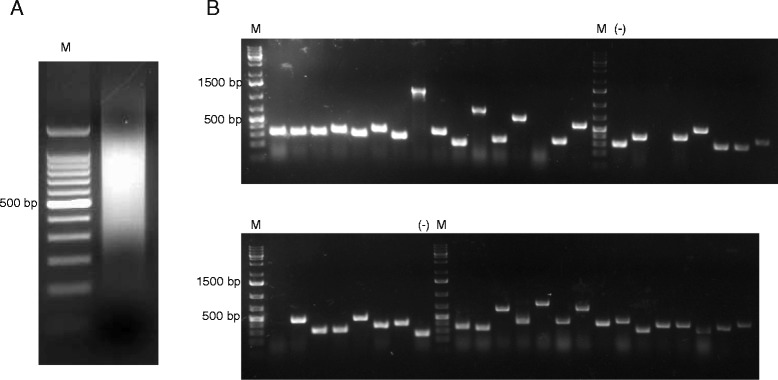
**a** Agarose gel electrophoresis of cDNA obtained after SMART cDNA synthesis using mRNA from salivary glands of 18 h fed *I. scapularis* nymphs. cDNA was subsequently used to construct the antigen library. **b** Colony PCR from randomly picked clones after cloning salivary gland derived cDNA fragments into pHORF3 and transformation of *E. coli* Top10F’ to determine insert percentage and size distribution of the antigen library

In order to construct an antigen library containing proteins which are relevant in anti-tick immunity [2], the salivary glands of nymphal *I. scapularis* previously fed for 18 h on a rodent host were dissected and mRNA was extracted. This mRNA was reverse transcribed into double-stranded cDNA using the SMART cDNA synthesis Kit. In this protocol a PCR based amplification step is performed to obtain larger quantities of double-stranded cDNA of full-length transcripts from limited mRNA sources. The salivary gland derived cDNA obtained by this protocol revealed a size range from 100 bp to more than 2000 bp (Fig 1a). Subsequently, this cDNA was used to construct a phage display antigen library using the pHORF3/Hyperphage system [28, 30]. Since UTRs at the 5’ and the 3’ ends as well as stop codons are not desired for subsequent cloning into the phagemid vector pHORF3, a partial restriction enzyme digest using three blunt end cutters was performed (data not shown). Finally, cDNA fragments were ligated into pHORF3 and *E. coli* Top10F’ were transformed with this library. A Colony-PCR was performed to assess the range of insert sizes as well as the cloning efficiency. More than 90 % of the analyzed clones contained an insert ranging from 50 bp to 700 bp (Fig 1b). 20 clones were subsequently sequenced, verifying that inserts are containing tick salivary sequences (data not shown). Finally, the library diversity was determined to be 1 x 10^6^ independent clones as assessed by transformation efficiency.

Packaging of the library was performed using a helperphage lacking a functional gene encoding the pIII envelope protein called Hyperphage [30, 33]. Together with the pHORF3 phagemid, this system allows for ORF enrichment as only inserts which are in-frame with the pIII gene of the phagemid will be expressed as oligopeptide-pIII fusions and thus assemble infectious phage [28]. This library was packaged yielding a final phage titer of 2 x 10^11^ CFU/ml, ensuring sufficient coverage for each of the predicted 10^6^ antigens of the library.

### Analysis of human blood sera for SGH reactivity

For this study several human blood sera were obtained from donors who classified themselves as either tick-bite sensitive or tick-bite naive, the latter meaning that these individuals had never been bitten by a tick, according to their own statement. In contrast, tick-bite sensitive donors had been bitten multiple times and some reported strong and instant responses such as itching or rashes, which might also be triggered through humoral immunity (Table 1).

**Table 1:** Tick-bite status of the eight human blood donors used in this study. Donors were categorized according to their own statements to be either tick-bite naive (never bitten by a tick) or tick-bite sensitized (bitten by ticks in the past)

Donor number	Tick-Bite status
1	naive
2	sensitized
3	sensitized
4	sensitized
5	naive
6	naive
7	sensitized
8	sensitized

**Fig 2:**
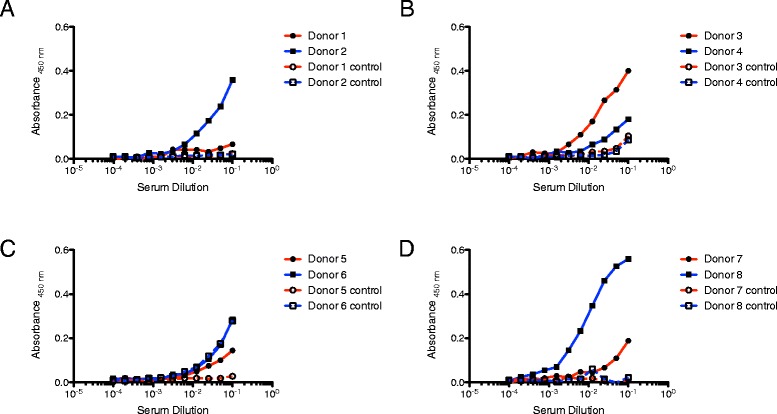
ELISA analysis to test the reactivity of serum IgG from eight different donors (**a**. donor 1+2, **b**. donor 3+4, **c**. donor 5+6, **d**. donor 7+8) to tick salivary gland homogenate (SGH). SGH was immobilized in the wells of a microtiter plate and screened with a dilution series for each serum (solid lines). Controls were performed with the same dilution series on blocked wells without SGH to determine unspecific background signals (dashed lines)

An ELISA was performed to analyze these blood sera for titers of antibodies recognizing tick salivary proteins. Salivary gland homogenate (SGH) of *I. scapularis* nymphs, fed for 18 h on a rodent host, was immobilized in the wells of a microtiter plate. Next, dilution series of human donor sera were tested for IgG antibodies recognizing the SGH. As a negative control the same sera dilution series were tested on blocked wells without SGH (Fig 2a-d). Note that even donors who classified themselves as tick-bite naive (i.e. donor 5) revealed some potential to recognize tick salivary proteins. On the contrary, some donors classified as tick-bite sensitive performed poorly in this assay (i.e. donor 4). According to this assay, donors 2 and 8 showed very promising results with respect to the presence of SGH specific immunoglobulins (Fig 2a and d).

### Enrichment of anti-SGH specific antibodies from human blood sera

Due to the complex nature of human blood sera, specific antibodies recognizing tick salivary proteins were purified from promising sera, as judged by the first ELISA analysis (Fig 2). This step was performed to increase the specificity and thus the significance of the screening of the phage display library (Fig 1) using highly concentrated and specific anti-SGH antibodies from tick-bite sensitive donors.

**Fig 3:**
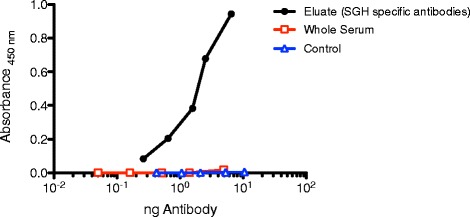
Representative experiment for enrichment of SGH specific antibodies from serum of donor 8. SGH-coupled carboxy beads were incubated with serum, washed and specifically binding antibodies were eluted. The eluted antibodies were quantified and tested for their reactivity to SGH in an ELISA (black curve). Serum was also incubated with control beads without coupled SGH and eluted antibodies were tested for reactivity with SGH in an ELISA (blue curve). Additionally, antibodies from whole serum without enrichment were tested (red curve)

For this purpose 18 h nymphal tick SGH was coupled to magnetic carboxy beads. These beads were used in a pull-down experiment with the sera of donors 2 (data not shown) and 8, which were judged as high performers according to Fig 2. After incubation with the sera, the beads were washed, and bound antibodies were eluted. The eluate was quantified and analyzed by ELISA for increased binding efficiency to SGH, compared to the original serum as well as a negative control eluate obtained from using blocked beads without SGH for the pull-down (Fig 3). The experiment confirmed the presence of highly specific IgG antibodies in the sera of donors 2 and 8 recognizing tick salivary proteins. Based on the confirmation of the presence of these antibodies, whole serum samples as well as enriched anti-SGH specific antibodies were used to screen our nymphal salivary gland library using phage display to identify proteins specifically recognized by these antibodies.

### Phage display screening for immunogenic nymphal tick salivary gland peptides

Immunogenic peptides from 18 h fed nymphal *I. scapularis* were identified by screening the phage antigen library against immunoglobulins of the IgG isotypes from human blood sera using the pHORF3 system [28]. Here, two different strategies were utilized. First, IgG from whole sera were captured and screened against the antigen library in three consecutive panning rounds. Second, the same procedure was followed using the anti-SGH specific antibodies previously enriched using the pull-down strategy instead of the whole sera.

**Fig 4:**
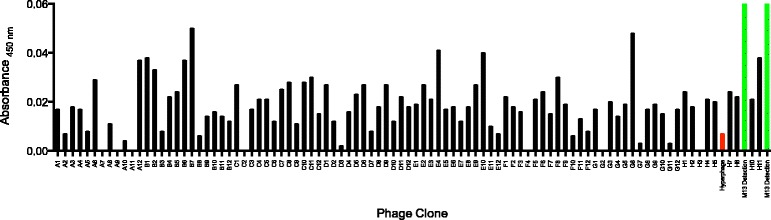
Result of a screening ELISA after three rounds of panning with SGH specific antibodies from donor 8. Monoclonal antigen-displaying phage were immobilized in the wells of a microtiter plate and screened for reactivity with the enriched serum antibodies. Letter-Number combinations on the x-axis represent the wells of the plate. Red bar represents immobilized Hyperphage, not displaying any peptide, as the negative control. Green bars represent detection of M13 phage as a positive control for phage immobilization

After three consecutive rounds of panning, a screening ELISA was performed using the resulting enriched phage clones to confirm the presentation of specifically recognized salivary peptides. Here, 92 clones were picked and monoclonal phages were produced in microtiter plates. These phage particles were immobilized in the wells of a microtiter plate and analyzed for their reactivity with IgG from the whole donor sera or enriched anti-SGH specific antibodies, respectively (Data not shown and Fig 4). As a negative control Hyperphage was immobilized and screened accordingly (Fig 4). Enriched peptides identified in this assay are summarized in Tables 2 and 3. Of note, these peptides are the result solely from screenings performed with sera from donors 2 and 8. Interestingly, screenings performed with sera from donors previously classified as tick-bite naive did not lead to the enrichment of any of the clones given in Table 2 (data not shown).

**Table 2:** Summary of immune-recognized peptides in this study. Information is given on the targets identified as well as the frequency at which they were found in the 27 clones above detection threshold after the third round of panning

Description	Accession Number	Frequency	Derived from Donor #
Ixodes scapularis conserved hypothetical protein, mRNA	XM_002402723.1	8/27	8
Ixodes scapularis solute carrier, putative, mRNA	XM_002416261.1	2/27	8
Ixodes scapularis thrombin inhibitor, putative, mRNA	XM_002408067.1	2/27	8
Ixodes scapularis ubiquitin protein ligase, putative, mRNA	XM_002412768.1	2/27	8
Ixodes scapularis salivary gland metalloprotease mRNA, complete cds	AY_264367.1	9/27	2 and 8
Ixodes scapularis 60S ribosomal protein L14, putative, mRNA	XM_002403042.1	2/27	2 and 8
Ixodes scapularis E3 ubiquitin protein ligase Bre1, putative, mRNA	XM_002434300.1	1/27	8
Ixodes scapularis DNA ligase, putative, mRNA	XM_002406537.1	1/27	2

**Table 3:** Amino acid sequences of the peptides displayed as fusions of pIII on M13 phage during panning and subsequent screening ELISA

Description	Accession Number	Peptide Sequence
Ixodes scapularis conserved hypothetical protein, mRNA	XM_002402723.1	LANVLVVATTVGVLHRVHGDTADLG
Ixodes scapularis solute carrier, putative, mRNA	XM_002416261.1	WD*IGERVGESLAVPSPTSCISCTSSSHFKHNSLFSGVLILGSVQVE
Ixodes scapularis thrombin inhibitor, putative, mRNA	XM_002408067.1	HDPDAVLFMGSIRE
Ixodes scapularis ubiquitin protein ligase, putative, mRNA	XM_002412768.1	SKPGTFWPAVSVPRCCSQGNR
Ixodes scapularis salivary gland metalloprotease mRNA, complete cds	AY_264367.1	KNMSEWVNGTLQSWTGGYAYVGTACSEWRVGMCEDRPT
Ixodes scapularis 60S ribosomal protein L14, putative, mRNA	XM_002403042.1	MRIPHSTSTKVVRR
Ixodes scapularis E3 ubiquitin protein ligase Bre1, putative, mRNA	XM_002434300.1	LPPSHRRVCTSVCDCGVHLRSQVLRVSPPRRTLLVPSKKRSCFECT
Ixodes scapularis DNA ligase, putative, mRNA	XM_002406537.1	LAKSDKDSVTHVLKGDHVKLK

### Validating the immunogenic character of the identified salivary proteins

Among the proteins identified (Table 2), four were selected for recombinant production in *E. coli* BLR(DE3) and subsequent validation of the immunogenic character in a titration ELISA assay with all donor sera. The proteins selected are *Ixodes scapularis* conserved hypothetical protein (XM_002402723.1) because it had the most significant enrichment throughout all screens, the thrombin inhibitor (XM_002408067.1) due to its potential role in interfering with host blood coagulation, the ubiquitin protein ligase (XM_002412768.1) since two molecules of this type were selected, and the metalloprotease (AY_264367.1) since this protease has been discussed in the context of tick immunity previously [34].

**Fig 5:**
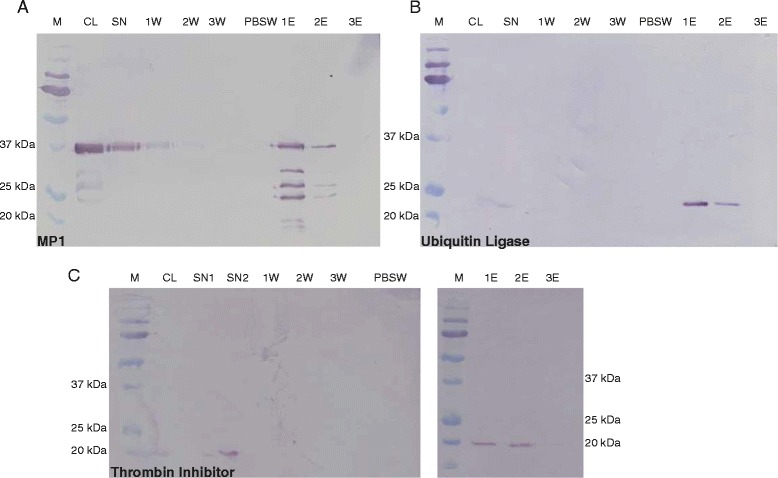
Western Blot analysis of recombinantly expressed tick salivary proteins metalloprotease 1 **a**, Ubiquitin Ligase **b** and Thrombin Inhibitor **c**. All three proteins were expressed as His-Tag fusion proteins and detected with an anti-His-Tag antibody. Abbreviations represent the following: CL crude lysate, SN supernatant after binding to Ni-Sepharose, 1 W first wash fraction, 2 W second wash fraction, 3 W third wash fraction, PBSW PBS wash fraction, 1E first eluted fraction, 2E second eluted fraction, 3E third eluted fraction

Subsequent cloning of the *I. scapularis* conserved hypothetical protein proved not possible. However, cloning of His-tagged versions of the other three proteins was successful, followed by their production in *E. coli* BLR(DE3) and subsequent purification (Fig 5).

**Fig 6:**
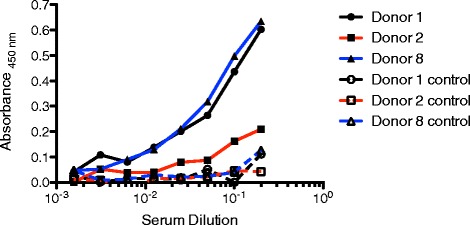
Titration ELISA to determine reactivity of IgG from donor sera with *I. scapularis* metalloprotease (MP). MP was recombinantly produced in *E. coli* BLR(DE3)*,* purified and immobilized in the wells of a microtiter plate. Reactivity of sera from donors 1, 2 and 8 is depicted (solid lines). As negative controls, sera were screened against blocked wells without recombinant MP (dashed lines)

Titration ELISA assays with dilutions of sera from all donors were performed for each of the purified proteins. While the thrombin inhibitor as well as the ubiquitin ligase did not reveal any specific reactivity to any donor serum as compared to a BSA negative control, the metalloprotease was clearly recognized by the sera from donors 1, 2 and 8 (Fig 6). Furthermore, sera from all other donors did not show specific binding of IgG antibodies compared to a BSA negative control (data not shown). This finding strongly suggests the immunogenic character of the metalloprotease and clearly shows the presence of specific antibodies in the sera from three donors, two of which self-reported as tick-bite sensitized.

## Discussion

Due to their ability to transmit pathogens, ticks pose significant risks to humans and domestic animals resulting in public health concerns and significant economic losses [1]. Current treatments to relieve tick burdens in endemic areas are mostly based on application of acaricides. This strategy, however, has negative impacts on the environment and agriculture, and raises concerns about the development of resistances [5]. Development of a broad-spectrum tick protective vaccine represents a promising alternative. Based on the phenomenon of acquired tick immunity [13], vaccination with molecules present in tick saliva appears to be a most sound public health approach [5]. The goal of this study was to identify *I. scapularis* salivary antigens that could serve as novel tick protective vaccine candidates for humans. A phage display approach was adapted to identify these proteins. The screening was designed to identify *I. scapularis* salivary antigens recognized by antibodies present in human blood sera.

This study reports the first use of antibodies from human blood sera as probes for detecting immunogenic salivary proteins. In former studies antibodies from rabbits were successfully used [20]. However, studies attempting to identify salivary proteins recognized by the human immune system are lacking. The prospect of utilizing human samples comes with some disadvantages compared to animal sera derived from controlled experiments. First, the availability of tick-naive serum donors is critical since donors reporting as tick-bite naive might have been unknowingly exposed to ticks. In contrast, tick-bite sensitized donors provide highly relevant sera. Importantly, the time interval since last tick exposure plays an essential role for the titers of specific antibodies in sera. This point likely explains the differences observed in IgG binding to salivary gland homogenate between individual donors in this study. The limitation of low antibody titers was overcome by enrichment of specific anti-SGH antibodies.

After establishing the screening protocol using human sera, eight unique oligopeptide phage clones were selected, showing significant reactivity to whole sera as well as enriched anti-tick specific antibodies from three tick-bite sensitized donors.

Among the proteins identified in this screening, the most promising candidate is metalloprotease 1 (MP1). MP1 has a theoretical molecular weight of 37 kDa, a conserved zinc-binding domain as well as twelve highly conserved cysteine residues. Apart from this protein being secreted into the extracellular space, MP1 has been described before as potentially immunogenic [34]. Therefore, these results confirm the suggested immunogenic character of MP1. Furthermore, MP1 has been stated to have fibrin(ogen)lytic activity as well as gelatinase activity [34]. Ticks could be utilizing these biochemical activities to counteract haemostasis or act in destruction of host extracellular matrix (ECM). Interestingly, it has been reported that *Borrelia burgdorferi* upregulates host metalloproteases in order to damage host ECM and facilitate infection [35]. This finding suggests a direct impact of tick feeding on pathogen transmission through compromising host defense. The selected oligopeptide of MP1 is located directly at the beginning of the cysteine-rich region and just 9 amino acids away from the zinc-binding domain. Accordingly, it is tempting to speculate that binding of an antibody to MP1 might have direct negative influence on the enzymatic function and/or enzyme-substrate interaction through steric hindrance.

If considering MP1 as a potential vaccine candidate, multi-species protection is of high interest [36]. Interestingly, MP1 has very close homologs in other tick species such as *I. pacificus* and *I. ricinus*, the latter being the most prevalent tick vector of Lyme borreliosis in Europe. Salivary gland metalloproteases identified in *I. ricinus* termed Metis (Metalloptotease from *Ixodes ricinus*) have been described previously and were shown to have good vaccination capacity. Furthermore, RNAi-based knock-down of Metis proteins in *I. ricinus* revealed increased tick mortality [37, 38]. Interestingly, sequence comparison of MP1 with Metis1 reveals 84 % identity between the two proteins on amino acid level. Despite having low homology to MP1 another vaccination study performed in rabbits using a metalloprotease from *Haemophysalis longicornis* also revealed good vaccination efficiency [39]. These findings, combined with the high degree of homology between Metis proteins and MP1, strongly favor MP1 as a putatively effective, multi-species vaccine candidate.

Besides metalloprotease MP1, the phage display strategy identified a putative thrombin inhibitor as another immunogenic tick salivary component. Unfortunately, the immunogenic character of this protein could not be confirmed via recombinant expression and ELISA. This shortcoming was potentially due to limitations in the bacterial expression system, including incomplete/improper folding or absence of post-translational modifications in the expressed protein. However, it is reasonable to speculate that a thrombin inhibitor provides a major benefit in tick feeding. Accordingly, counteracting the thrombin inhibitor through antibodies would be a sensible host response to tick feeding. Based on sequence similarity, the identified protein can be placed in the serpin family of protease inhibitors, suggesting its ability to interfere with the blood clotting cascade and other protease cascades involved in inflammation such as complement activation or catabolism of phagocytosed proteins. Interestingly, a recent publication identifies a thrombin inhibitor from *I. scapularis,* which is reported to inhibit not only thrombin but also Factor Xa and cathepsin G, revealing activities against haemostasis and inflammatory responses [40]. This report bolsters significance and credibility to the thrombin inhibitor identified in this screen as a potential vaccine target.

Excluding the metalloprotease and the thrombin inhibitor mentioned previously, all other binders identified in this work are intracellular proteins. While it may seem counterintuitive to envision intracellular proteins as potential vaccine candidates, just such a tick ribosomal protein has recently been suggested [41]. The well documented, dynamic nature of salivary gland protein expression during feeding [11, 42] necessitates a synchronization between cell proliferation and cell death; dying cells can leak intracellular components into the surrounding fluids, thus enabling host contact and initiation of the adaptive immune response. Antibodies binding these displaced intracellular proteins may form immune complexes at the tick-bite site and help recruit other cells of the immune system such as neutrophils or macrophages [17], thereby providing a mechanism for inducing inflammation at a site that is otherwise strongly immune-suppressed through the activities of many other of the tick salivary proteins. Of note, a recent study using a T7 phage display based approach to identify the tick saliva immune-proteome reports a broad range of proteins, secreted and intracellular, to be putatively immunogenic [43].

The discovery of Metalloprotease 1 appears highly promising due to its published recognition as an immunogenic antigen and the high efficiency of other tick metalloproteases in stimulating a vaccination response [34, 37–39]. All identified proteins in this study represent potentially important new tools in solving the tick problem; they may prove not only for their strong potential as anti-tick vaccine candidates but also for diagnostic purposes as biomarkers of vector tick exposure.

## Conclusion

In this work, MP1 of *I. scapularis* was identified as immunogenic protein. This protein was hypothesized as immunogenic in former publications. Because MP1 is highly homologous in all *Ixodes* species, this protein is an interesting biomarker and potential candidate for vaccine development.

## Materials and Methods

### Tick rearing and salivary gland homogenate

*Ixodes scapularis* ticks were reared as described previously [44]. Adult ticks were collected from nature to create immature tick colonies. One hundred larvae from each egg batch were screened by PCR using pathogen-specific primers for *Borrelia burgdorferi* and *Borrelia miyamotoi.* Certified pathogen-free larval stage ticks were blood fed on hamsters or white-footed mice to promote molting. All unfed nymphal ticks were maintained at 23 °C and >90 % relative humidity under 14 h light/10 h dark photoperiod before infesting hosts. Methods for generating uninfected tick colonies, including animal care, followed protocols approved by the Institutional Animal Care and Use Committee (IACUC) at the the University of Rhode Island (URI). Comprised of faculty members, an Attending Veterinarian, and at least one representative of the public (not affiliated with URI), the IACUC oversees all URI research and instruction that involves vertebrate animals, in order to ensure that the highest ethical and animal welfare standards are met. URI IACUC has approved all animal use protocols for generating uninfected ticks, ensuring compliance with federal regulations. Additionally, IACUC has inspected all animal facilities and laboratories in which this research was completed. All URI researchers are trained and certified yearly to ensure compliance with federal regulations regarding animal welfare. Pathogen-free nymphs were allowed to feed for 18 h prior to dissection for salivary glands. Partially-fed ticks were dissected in ice-cold phosphate buffered saline (PBS) [45] within 4 h of being removed from the host animal. After removal, glands were washed in the clean buffer and tissues were stored at −70 °C in PBS until cell lysis by sonication. Protein concentration was detected by UV nanodrop quantification (ThermoScientific, ND-1000).

### mRNA isolation from salivary gland and SMART cDNA synthesis

mRNA from tick salivary glands was isolated using the Illustra mRNA purification Kit (GE Healthcare, Munich, Germany) according to the supplier’s instructions. Subsequently, the mRNA obtained was further concentrated using the RNeasy Mini Elute clean-up Kit (Qiagen, Hilden, Germany) according to the supplier’s instructions.

Tick salivary gland derived mRNA was used to generate double-stranded cDNA. 13 μl mRNA (at least 25 ng) were mixed with 1 μl 3′ SMART CDS Primer II A (12 μM) (5′ AAGCAGTGGTATCAACGCAGAGTACT(30)AT 3′) and 1 μl SMART II A Oligonucleotide (12 μM) (5′ AAGCAGTGGTATCAACGCAGAGTACGCGGG 3′). The mixture was incubated at 72 °C for 2 min and placed on ice for 2 min. Subsequently, 4 μl 5x First Strand Buffer (Life Technologies, Darmstadt), 0.2 μl DTT, 1 μl dNTP Mix (10 mM each) and 1 μl M-MLV reverse transcriptase (Life Technologies, Darmstadt) were added and the mixture was incubated at 37 °C for 1 h yielding first strand cDNA. For second strand synthesis a long distance PCR was performed using 2 μL first strand cDNA, 10 μL ExTaq buffer (Takara), 4 μL dNTP mix (2.5 mM each), 2 μL 5′ PCR Primer IIA (12 μM) (5′ AAGCAGTGGTATCAACGCAGAGT 3′), 0.25 μL ExTaq DNA Polymerase (Takara), 36.75 μL dH_2_O. The PCR reaction was performed for 24 cycles (15 s 95 °C, 30 s 65 °C, 6 min 72 °C) followed by a 10 min final synthesis step. The PCR products were purified using the Nucleospin Extract 2 Kit (Macherey-Nagel, Düren, Germany).

### Salivary gland library construction

To generate blunt ended DNA fragments suitable for cloning into pHORF3 [28] a restriction enzyme digest using the blunt end cutting enzymes AfeI, AluI and CviKI-1 (NEB, Frankfurt, Germany) was performed. 30 μl SMART cDNA (~1.7 μg) were mixed with 1.25 U of each of the restriction enzymes, 10 μL NEBuffer 4 (NEB), 10 μL 10xBSA solution (NEB) in a total volume of 100 μl. The digestion was incubated at 37 °C for 10 min followed by a heat inactivation at 65 °C for 20 min.

The phagemid vector pHORF3 was linearized by digestion with PmeI (NEB). Incubation was performed at 37 °C over night followed by heat inactivation at 65 °C for 10 min. Subsequently, the vector was dephosphorylated with 0.5 μl calf intestinal phosphatase (CIP) (NEB) at 37 °C for 30 min. Linearized and dephosphorylated vector was purified using the NucleoSpin Kit (Machery Nagel).

For library cloning, a 10x fold molar excess of cDNA was cloned into 500 ng of linearized pHORF3. The ligation was performed in 60 μL volume with 3U T4 DNA Ligase (Promega, Mannheim, Germany) at 16 °C over night. Ligation mixture was added to 50 μl electrocompetent *E. coli* TOP 10 F’ cells (Life Technologies) and incubated on ice for 5 min. Subsequently the DNA-cell suspension was filled into pre-chilled cuvettes and electroporation was performed with a pulse of 1.7 kV. 1 ml SOC [45] medium with a temperature of 37 °C was added immediately and the cells were incubated at 37 °C and 600 rpm for 1 h. Finally, the whole cell suspension was plated on 2xYT agar [45] plates supplemented with 0.1 M glucose and 100 μg/mL ampicillin. Plates were incubated over night at 37 °C. Colonies were floated off the agar plates with 40 ml 2xYT medium. The resulting cell suspension was centrifuged at 3220 x g, 4 °C for 15 min. The pellet was resuspended in 2xYT medium containing 20 % glycerol and the sample was stored at −80 °C.

### Library packaging

400 ml 2xYT medium supplemented with 0.1 M glucose and 100 μg/mL ampicillin (2xYT-GA) were inoculated with 1 ml antigen library glycerol stock and bacteria were grown at 37 °C and 250 rpm until OD 600 = 0.5. 25 ml from that culture were transferred to 50 ml reaction tubes and infected with 2.5 x 10^11^ CFU Hyperphage [30, 31, 33]. Infection was achieved by incubating at 37 °C for 30 min followed by a second incubation for 30 min at 37 °C and 250 rpm. After centrifugation at 3220 x g for 10 min the cell pellet was resuspended in 400 ml 2xYT supplemented with 100 μg/mL ampicillin and 50 μg/mL kanamycin (2xYT-AK) and the culture was incubated 24 h at 30 °C and 250 rpm. The cell suspension was centrifuged at 6000 x g, 4 °C for 20 min and the supernatant was transferred into a fresh tube. For precipitation of the phage 1/5 volume PEG/NaCl (20 % (w/v), 2.5 M NaCl) was added to the suspension which was subsequently incubated at 4 °C over night. Phage were pelleted by centrifugation at 10,000 x g, 4 °C for 1 h. The supernatant was discarded and the pellet was resuspended in 20 ml phage dilution buffer (10 mM Tris, 20 mM NaCl, 2 mM EDTA) and filter-sterilized (pore size 0.45 μm). Again 1/5 volume PEG/NaCl was added and the sample was incubated on ice for 1 h followed by a centrifugation at 20,000 x g, 4 °C for 20 min. The resulting pellet was resuspended in 5 ml phage dilution buffer and the oligopeptide phage particles were stored at 4 °C.

### Enrichment of serum antibodies against salivary gland homogenate (SGH)

The study was performed in accordance with the Declaration of Helsinki and was conducted in accordance with University of Rhode Island’s Institutional Review Board, Approval number HU1011-041. All voluntary donors were informed about the project and gave their informed consent. To enrich serum antibodies specific for salivary gland homogenate (SGH) 2 x 10^7^ Carboxy Beads (Dynabeads, Life Technologies) were washed twice with 1 ml PBST (PBS, 0.05 % Tween20) and twice with 0.5 ml NaAc buffer (pH 4.5) by rotating for 5 min. Next, 100 μl EDC and 100 μl NHS were added and the beads were rotated for 10 min at room temperature followed by another two washing steps with 0.5 ml NaAc buffer (pH 4.5) [45] and 5 min of rotation each. 20 μg SGH (1 μg/10^6^ beads) were diluted in 200 μl NaAc buffer and incubated with the beads under rotation for 20 min at room temperature. Subsequently, the beads were washed three times with 1 ml PBST followed by a 2 h rotation with 1 ml 100 mM ethanol amine. After this blocking step the beads were washed three times with 1 ml PBST, once with 1 ml PBS followed by an equilibration in 1 ml 0.1 M glycine/HCl pH 2.2. The beads were washed another three times with 1 ml PBS and incubated with 400 μl of a 1:1 serum-PBS dilution under rotation at 4 °C over night. Next, the beads were washed three times with 1 ml PBS and the serum antibodies were eluted by incubation with 150 μl 0.1 M glycine/HCl pH 2.2 under rotation for 15 min at room temperature. The supernatant was neutralized by addition of 45 μl 0.5 M Tris–HCl pH 8 and brought to a total volume of 300 μl by adding PBS. The eluted antibodies were stored at 4 °C.

### Selection of immunogenic oligopeptides (panning)

For selection of immunogenic oligopeptide-phage mouse α-human IgG (Fc specific) (I6760, Sigma, Munich, Germany) monoclonal antibody was diluted 1:5,000 in carbonate buffer [45] and 100 μl were immobilized in a Costar polystyrole microtitre plate (MTP) well (Corning, Germany) at 4 °C over night. In parallel, 50 μL SGH enriched antibody fraction and 1x10^11^ Hyperphage particles were pre-incubated at 4 °C over night in 150 μL PBST supplemented with 2 % (w/v) skim milk powder (2 % MPBST). The MTP well with the capture antibody was blocked with 350 μl 2 % MPBST for 1.5 h at room temperature followed by three washing steps with PBST using an ELISA washer. The pre-incubated patient serum was transferred into the MTP well with the blocked capture antibodies and incubated at room temperature for 2 h followed by three washing steps with PBST. 200 μl oligopeptde library phage or phage from the previous panning round were filled into the MTP well and incubated for 2 h at room temperature. This incubation was followed by 10 x N stringent washing steps with PBST where N = number of the panning round. Bound phage particles were eluted by addition of 200 μl Trypsin solution (10 μg/ml in PBS) and incubation at 37 °C for 30 min. 190 μl of the eluted phage were used to re-infect *E. coli* TOP10F’ for phage amplification. The remaining 10 μl were used for titration of the eluted phage.

For the amplification of eluted phage 50 mL 2xYT-T medium were inoculated with 200 μl of an *E. coli* TOP10F’ starter culture and the culture was grown at 37 °C and 250 rpm to OD_600_ = 0.5. The eluted phage were added to 20 mL of the culture and incubated at 37 °C for 30 min followed by a second incubation for 30 min at 37 °C and 250 rpm. The re-infected cells were pelleted by centrifugation at 3220 x g for 10 min. The pellet was re-suspended in 250 μl 2xYT medium, plated on a 15 cm 2xYT-GA agar plate and incubated at 37 °C over night. Colonies were floated off the agar plate using 5 ml 2xYT medium. 250 μl of the cell suspension were used to inoculate 50 ml 2xYT-GA medium to OD_600_ = 0.05 to 0.09. The culture was subsequently grown at 37 °C and 250 rpm to OD_600_ = 0.5. 5 ml culture were infected with 5 x 10^11^ Hyperphage particles by incubation at 37 °C for 30 min followed by a second incubation for 30 min at 37 °C and 250 rpm. The cells were pelleted at 3220 x g for 10 min and the pellet was resuspended in 30 ml 2xYT-AK followed by an incubation at 30 °C and 250 rpm overnight. Produced oligopeptide phage were pelleted as outlined above. The phage pellet was re-suspended in 500 μl phage dilution buffer and stored at 4 °C until it was used in the next panning round. 10 μl were used to titer the amplified phage. In each selection three rounds of panning were performed.

### Screening Elisa

To produce monoclonal oligopeptide phage particles, polypropylene MTPs (96 Well, flat bottom, Sarstedt, Germany) were filled with 150 μl 2xYT-GA medium and each well was inoculated with a single colony resulting from the third panning round and incubated over night at 37 °C and 300 rpm. A new MTP was filled with 150 μl 2xYT-GA medium and each well was inoculated with 10 μl over night culture from the respective well of the master MTP and incubated for exactly 2 h at 37 °C and 300 rpm. Next, 5 x 10^9^ Hyperphage particles were added per well and the MTP was incubated at 37 °C for 30 min, followed by a second 30 min incubation at 37 °C and 300 rpm. Subsequently, bacteria were pelleted by centrifugation at 3220 x g for 10 min and the supernatant was discarded. The pellets were re-suspended in 150 μl 2xYT-AK and the MTP was incubated at 37 °C and 300 rpm over night. On the next day, the MTP was centrifuged at 3220 x g for 10 min and the phage supernatants were transferred to a new polypropylene MTP. 40 μl PEG/NaCl were added to each well and the plate was incubated on ice at 4 °C over night. Phage particles were pelleted by centrifugation at 3220 x g for 1 h at 4 °C. The supernatants were discarded and the phage pellets were re-suspended in 150 μl phage dilution buffer. The plate was centrifuged again at 3220 x g for 10 min to pellet remaining bacteria and the supernatants were transferred to a new polypropylene MTP.

For the screening ELISA, mouse α-M13 capture antibody (B62-FE2, Progen, Heidelberg, Germany) was diluted 1:400 in 100 μL carbonate buffer and incubated in Costar MTP wells over night at 4 °C. In parallel, 50 μL serum, 1x10^11^ Hyperphage particles were pre-incubated at 4 °C over night in 100 μL 2 % MPBST. The wells were blocked with 2 % MPBST for 1.5 h at room temperature followed by three washing steps with PBST using an ELISA washer. 50 μl monoclonal oligopeptide phage and 50 μl 2 % MPBST were transferred into the well with the anti-M13 capture antibody and incubated at room temperature for 2 h. As negative control 1x10^10^ Hyperphage were used. After three washing steps with PBST using an ELISA washer 100 μl pre-incubated serum were filled into each well and incubated for 2 h at room temperature followed by three washing steps with PBST. Bound human serum antibodies were detected with goat α-human IgG (Fc specific) conjugated with horseradish peroxidase (A0170, Sigma) diluted 1:70,000 in 2 % MPBST for 1 h at room temperature. As positive control mouse α-M13 HRP conjugate antibody (GE Healthcare) diluted 1:40,000 in 2 % MPBST was used. Visualization was performed with TMB (3,3′,5,5′-tetramethylbenzidine) substrate. The staining reaction was stopped by adding 100 μL 1 N sulphuric acid. The absorbance at 450 nm and scattered light at 620 nm were measured and the 620 nm value was subtracted using a SUNRISE microtiter plate reader (Tecan, Crailsheim, Germany).

### Cloning of metalloprotease 1, ubiquitin ligase and thrombin inhibitor

The cDNA of the proteins was amplified by PCR using the following oligonucleotide primers: for the metalloprotease 1 MHMetalI_f1 (5′ gcgtg gctagc cat cat cat cac cat cac agc tac aag atc ccc ttg g 3′) and MHMetall_r1 (5′ cgcac gcggccgc tca tta gtc atc ttt gct tat ttt gat atc g 3′), for the ubiquitin protein ligase ANFubiprotlig_f2 (5′ gcgtg gctagc gag gcg cag tac aac ctc c 3′) and ANFubiprotlig_r1 (5′ cgcac gcggccgc ctt aaa agt gat ttg tgc agc 3′) and for the thrombin inhibitor ANFthrominhi _f2 (5′ gcgtg gctagc cac cag gaa ggg ga ctt caa gat ggg 3′) and ANFthrominhi _r1 (5′ cgcac gcggccgc gag ctc acg gat gga tcc c 3′). The PCR product of the metalloprotease 1 was cloned into pET21A+ and the PCR products of the ubiquitin protein ligase and the thrombin inhibitor were cloned into pET21A + −pelB, using NheI and NotI restriction sites. After ligation the plasmids were transformed into *E. coli* BLR-DE3. Positive clones were identified by colony PCR using the oligonucleotide primers MHpET21_f1 (5′ GAGCGGATAACAATTCCCC 3′) and MHpET21_r1 (5′ GCAGCCAACTCAGCTTCC 3′).

### Production of metalloprotease 1 in *E. coli*

250 mL 2xTY-GA medium were inoculated with 5 mL overnight culture and cultivated to an OD_600_ = 0.8 at 37 °C and 250 rpm. The expression was induced with 1 mM IPTG (final concentration) and incubated for 4 h at 22 °C and 220 rpm. Cells were harvested by centrifugation at 4,400 x g for 15 min and 4 °C. Lysis was performed with 1 mg/mL lysozyme and 5 μg/mL DNAseI in 20 mL His-tag binding buffer pH8.0 (20 mM Na_2_HPO_4_, 0.5 M NaCl) supplemented with 10 mM Imidazole for 15 min at 30 °C followed by 4 min sonication (40 % power, 30 s pulse, 30 s pause) (Sonotrode MS72, Bandelin, Berlin, Germany). The suspension was centrifuged for 15 min at 4 °C and 27,000 x g. The pellet was washed twice with His-tag binding buffer supplemented with 10 mM Imidazole and 0.5 % Triton X-100. Finally, the pellet was resuspended in His-tag binding buffer, supplemented with 8 M urea and centrifuged for 30 min at 4 °C and 27,000 x g. The His-tagged metalloprotease in the supernatant was purified under denaturing conditions with FastFlow Sepharose (GE Healthcare) loaded with nickel. The Sepharose was washed with 10 mM, 30 mM and 60 mM imidazole (20 mM Na_2_HPO_4_, 0,5 M NaCl, 10, 30 or 60 mM Imidazol). For elution, 5 mL 100 mM EDTA in PBS supplemented with 8 M urea were used. The purified proteins were stored at −20 °C.

### Production of ubiquitin ligase and thrombin inhibitor in *E. coli*

250 mL 2xTY-GA medium were inoculated with 5 mL overnight culture and cultivated to an OD_600_ = 0.8 at 37 °C and 250 rpm. The expression was induced with 1 mM IPTG (final concentration) and incubated for 4 h at 22 °C and 220 rpm. Cells were harvested by centrifugation at 4,400 x g for 15 min and 4 °C. The Pellet was resuspended in 25 ml ice cold PE buffer (500 mM Succrose, 100 mM Tris pH8.0, 1 mM EDTA) and incubated on ice for 20 min with stirring or shaking. The suspension was centrifuged for 30 min at 4 °C and 27,000 x g. The supernatant (periplasmatic preparation (PPP)) was stored on ice. The pellet was resuspended in 25 ml ice cold 5 mM Mg_2_SO_4_ and incubated on ice for 20 min with stirring or shaking. The suspension was centrifuged for 30 min at 4 °C and 27,000 x g. The supernatant (osmotic shock preparation (OSP)) was pooled with the PPP. His-tag binding buffer pH 8.0 (20 mM Na_2_HPO_4_, 0.5 M NaCl) supplemented with 10 mM Imidazole was added 1:1 to the pooled fraction. The proteins were purified with FastFlow Sepharose (GE Healthcare) loaded with nickel. The Sepharose was washed with 10 mM, 20 mM and 30 mM imidazole (20 mM Na_2_HPO_4_, 0,5 M NaCl, 10, 20 or 30 mM Imidazol). For elution, 5 mL 100 mM EDTA in PBS was used. The purified proteins were stored at −20 °C.

### Titration ELISA

200 ng metalloprotease 1 were coated to 96 well microtiter plates (MaxiSorp, Nunc) in 50 mM NaHCO_3_ pH 9.6 overnight at 4 °C. In parallel, human sera were pre-incubated in PBST supplemented with 2 % (w/v) BSA. After coating, the wells were washed three times with PBST and blocked with 2 % MPBST for 1.5 h at RT, followed by three washing steps with PBST. Human sera were diluted in 100 μL 2 % MPBST and incubated in the metalloprotease 1 coated plates for 2 h at RT, followed by three PBST washing cycles. Bound human IgGs were detected with goat anti-human IgG HRP conjugate (1:78,000 in 2 % MPBST) (A0170, Sigma). The visualization was performed with TMB as described above.

## Abbreviations

ATR: Acquired tick immunity
bp: Base pair
BSA: Bovine serum albumin
cDNA: Complementary deoxyribonucleic acid
DNA: Deoxyribonucleic acid
ECM: Extracellular matrix
ELISA: Enzyme-linked immunosorbent assay
IgE: Immunoglobulin E
IgG: Immunoglobulin E
kDa: Kilo Dalton
mRNA: Messenger ribonucleic acid
MP1: Metalloprotease 1
ORF: Open reading frame
PCR: Polymerase chain reaction
RNAi: Ribonucleic acid interference
SGH: Salivary gland homogenate
UTR: Untranslated region
